# Red blood cell to platelet ratio and its trajectory as prognostic factors for patients with gastrointestinal tract perforation and abdominal sepsis

**DOI:** 10.1371/journal.pone.0337480

**Published:** 2025-12-05

**Authors:** Jie Zhao, Junkun Zhang, Jixiu Fan, Heihei Li, Weidong Wu, Jifang Liang

**Affiliations:** 1 Shanxi Bethune Hospital, Shanxi Academy of Medical Sciences, Tongji Shanxi Hospital, Third Hospital of Shanxi Medical University, Taiyuan, China; 2 Tongji Hospital, Tongji Medical College, Huazhong University of Science and Technology, Wuhan, China; 3 Central Hospital of China Railway Bureau Group Company Limited, Taiyuan, Shanxi Province, China; 4 Shanxi Bethune Hospital, Shanxi Academy of Medical Sciences, Tongji Shanxi Hospital, Third Hospital of Shanxi Medical University, Taiyuan, China; 5 Third Hospital of Shanxi Medical University, Shanxi Bethune Hospital, Shanxi Academy of Medical Sciences, Tongji Shanxi Hospital, Taiyuan, China; Children's National Hospital, George Washington University, UNITED STATES OF AMERICA

## Abstract

**Background:**

Sepsis after emergency gastrointestinal perforation surgery increases costs and hospital stay. Early diagnosis and management are vital. This study assesses the prognostic value of the red blood cell to platelet ratio (RPR) in these patients with abdominal sepsis.

**Method:**

Clinical data from MIMIC-IV and a hospital validation set were retrospectively analyzed. Demographics, comorbidities, and lab indices were extracted; missing values were imputed using random forest. RPR’s dynamic changes and relation to prognosis were analyzed using latent category trajectory modeling. Prognostic factors were screened via Lasso-Cox regression to build column-line graph models. Kaplan-Meier, log-rank tests, and restricted cubic splines analyzed RPR-outcome associations. R 4.2.3 was used; P < 0.05 was significant.

**Results:**

A total of 243 patients were enrolled in MIMIC-IV and 253 patients were enrolled in the hospital. RPR was a significant prognostic indicator. Elevated RPR correlated with coagulation dysfunction (increased PT/INR, decreased fibrinogen) and higher mortality risk. Trajectory analysis identified two RPR patterns; Class 1 had significantly lower 28-day mortality than Class 2. The hospitalization and ICU prognostic models demonstrated good efficacy. Validation set results supported these findings, indicating dynamic RPR changes effectively assess prognosis.

**Conclusions:**

The red blood cell to platelet ratio may serve as a novel prognostic biomarker for abdominal septic patients, influencing clotting and kidney function.

## Introduction

Gastrointestinal (GLT) perforation is a life-threatening clinical emergency most commonly caused by conditions such as peptic ulcer disease, diverticulitis, trauma, malignancy, intestinal ischemia, inflammatory bowel disease, or severe foodborne infections [1–3]. The common pathological outcome is a loss of gastrointestinal wall integrity, leading to the leakage of contents into the peritoneal cavity, which triggers diffuse peritonitis and often rapidly progresses to life-threatening abdominal sepsis and multiple organ failure, with an overall mortality rate ranging from 30% to 50% [4]. Given that the mortality rate for sepsis in postoperative patients can reach 50% [5,6], the early recognition of gastrointestinal perforation and the active management of sepsis have become crucial for improving patient outcomes.

Despite, we have achieved significant advances in antibiotic drugs and therapeutic methods, the hospital mortality rate of abdominal sepsis remains high. The mortality of postoperative sepsis among patients with GIP accounts to 50% [7–13]. Therefore, postoperative sepsis is a severe complication, early diagnosis and effective treatment can ameliorate patient prognosis and reduce mortality [14]. It is well known that bacterial culture remains the gold standard for detecting sepsis pathogens, but its time-consuming nature leads to diagnostic delays and may yield false-negative results in real-world infections [15–17]. Emerging rapid detection technologies, such as the Metabolism-Driven Colorimetric Sensor Array, can address this gap—recent studies confirm this technology can complete bacterial detection and antimicrobial susceptibility testing within hours [18]. However, achieving high sensitivity and specificity in complex clinical samples remains a barrier to its widespread adoption. Sepsis is a diverse disease state that is prevalent and significant in critically ill patients. Stratifying patients into more homogeneous groups with similar biological characteristics may help in finding new therapies [19].

Red blood cells, especially smaller subgroups, appear to be highly susceptible to damage caused by sepsis, providing early warning signals of sepsis and contributing to microvascular dysfunction associated with organ dysfunction [20]. During sepsis, reduced red blood cell hemoglobin content may lead to microcirculatory dysfunction and immune dysregulation [21,22]. During sepsis, platelets as cellular mediators of thrombosis are activated and increasingly recognized as mediators of immune responses [23].Platelet changes accompanying sepsis are associated with mortality [24]. Although studies have shown that platelets and red blood cells have distinct functions in inflammation and immune response, there is limited research that has truly explored the shared functions of platelets and red blood cells.

Recent studies have reported a significant association between low red blood cell to platelet ratio (RPR) levels and adverse outcomes in traumatic patients [25]. The predictive value of RPR for clinical endings in patients with abdominal sepsis remains uncertain. This study aimed to explore the association between RPR and clinical outcomes in patients with abdominal sepsis, aiming to develop an convenient predictor to assess patients’ prognosis.

## Methods

### Study population

In this study, the authors retrospectively retrieved data of patients with gastrointestinal perforation complicated by sepsis from a large database called MIMIC-IV developed and managed by the Massachusetts Institute of Technology (MIT) Laboratory for Computational Physiology [26]. This database contains medical information of intensive care unit (ICU) admissions at Beth Israel Deaconess Medical Center. One of the authors of this study was granted access to this dataset and extracted relevant data. The use of this database was approved by the institutional review boards of MIT and Beth Israel Deaconess Medical Center for research purposes, and informed consent was waived. The diagnoses of gastrointestinal perforation and sepsis in the study were based on International Classification of Diseases (ICD-9 and ICD-10) codes (all ICD-9 and ICD-10 codes for diseases are listed in S1 Table). In this study, we included patients with gastrointestinal perforation complicated by sepsis, excluded samples with incomplete laboratory and biochemical indicators, and finally included 243 cases.

This study is a single-center retrospective validation cohort. Data were sourced from patients with gastrointestinal perforation complicated by sepsis who were admitted to Shanxi Bethune Hospital between 01/01/2022 and 31/05/2024. The data were accessed for research purposes on 01/09/2024. Throughout the study, researchers had no access to any information that could identify individual participants. Ethical approval for this study was granted by the Ethics Committee of YXLL-2023–269. All data were anonymized prior to analysis to ensure patient privacy protection. Data were collected through the hospital’s electronic medical record system, which provided demographic characteristics, laboratory indicators, and prognosis data. A total of 253 patients who met the inclusion criteria were included in this study as the external validation cohort, which will be used to further evaluate the clinical applicability of the model.

### Data extraction and preprocessing

In this study, information was extracted through running Structured Query Language (SQL) using PostgresSQL (version 13.7.2) and Navicate Premium (version 16) software. Potential confounding variables included in this study are as follows: 1. Baseline demographic information: age, gender, weight; 2. Comorbidities: Hypertension, Diabetes, Malignant tumor, Hyperlipidemia, Chronic bronchitis; 3. Laboratory parameters: WBC, RBC, Platelet, Hemoglobin, Glucose, Total bilirubin, Creatinine; 4. Blood gas indicators: PH, Pco2, Po2, Spo2, Sodium, Potassium, Total calcium; 5. Coagulation indicators: PTT, INR.. Considering that missing values did not exceed 5%, we used the random forest method for multiple imputation of all serum indicators.

### Definition of exposure variables and outcome events

Based on the detection indicators RBC and Platelet used in this study, the red blood cell to platelet ratio was calculated for each patient. The primary outcome events in this study were all-cause mortality within 365 days after patient admission and within 365 days after ICU admission, while the secondary outcome events were defined as all-cause mortality within 28 days after admission and within 28 days after ICU admission.

### Red blood cell to platelet ratio trajectory analysis

Statistical analysis for this study was conducted using R studio (version R4.2.3). After obtaining the red blood cell to platelet ratio for the first four days of patients, this study used the lcmm package and latent class trajectory models to identify the trajectories of red blood cell to platelet ratio over time [27]. The model fits trajectories for 1–4 classes, and the optimal model is selected based on the minimum value of the Bayesian Information Criterion (BIC). The prognostic differences between different trajectory groups are compared using Kaplan-Meier survival curves and the log-rank test.

Restricted cubic spline (RCS) curves were employed to explore the association between the red blood cell to platelet ratio and outcome events, and a threshold effect model was developed to analyze the inflection point of the red blood cell to platelet ratio [28].

### Construction of the prognostic model

The glmnet package in R was used to integrate data on survival time, survival status, and clinical variables. Least Absolute Shrinkage and Selection Operator (LASSO) regression analysis was conducted, with λ.min selected as the optimal λ value to identify key predictive variables. Subsequently, the variables selected by LASSO were incorporated using the rms package to construct a prognostic nomogram based on the Cox proportional hazards model.

### External validation

This study was externally validated using an independent single-center clinical dataset. This external validation cohort comprised patients hospitalized between 01/01/2022 and 31/05/2024 for gastrointestinal perforation complicated by sepsis. The inclusion and exclusion criteria for the validation set were consistent with those of the training set (MIMIC-IV). Baseline characteristics, variable definitions, and methods for handling missing data were consistent with those of the training set. The model’s predictive performance was evaluated by assessing the clinical outcomes of patients using ROC curves.

## Results

### The ratio of red blood cell count to platelet count affects the prognosis of patients with gastrointestinal perforation complicated by sepsis

This study included 243 patients with gastrointestinal perforation complicated by sepsis. Subsequently, the data of red blood cell and platelet counts in the 4 days prior to ICU admission were used to calculate the ratio of red blood cells to platelets to obtain the ratio of red blood cells to platelets in the first four days. The two-class trajectory model, which has the lowest BIC, is considered the optimal model.

**Fig 1:**
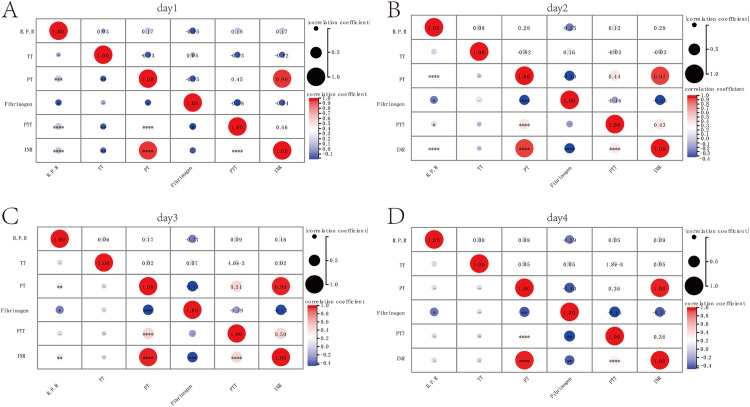
Correlation analysis of red blood cell-platelet ratio and coagulation indicators. **(A)** Scatter plot of correlation between red blood cell-platelet ratio and coagulation indicators on the first day in the ICU. **(B)** Scatter plot of correlation between red blood cell-platelet ratio and coagulation indicators on the second day in the ICU. **(C)** Scatter plot of correlation between red blood cell-platelet ratio and coagulation indicators on the third day in the ICU. **(D)** Scatter plot of correlation between red blood cell-platelet ratio and coagulation indicators on the fourth day in the ICU.

Through correlation analysis, it was found that on the first day in the ICU, the red blood cell-platelet ratio was positively correlated with PT (cor = 0.17), PTT (cor = 0.18), and INR (cor = 0.17), and negatively correlated with Fibrinogen (cor = −0.15) (Fig 1A). On the second day in the ICU, the red blood cell-platelet ratio was positively correlated with PT (cor = 0.28), PTT (cor = 0.12), and INR (cor = 0.29), and negatively correlated with Fibrinogen (cor = −0.25) (Fig 1B). By the third day in the ICU, the red blood cell-platelet ratio was positively correlated with PT (cor = 0.17) and INR (cor = 0.16), and negatively correlated with Fibrinogen (cor = −0.27) (Fig 1C). On the fourth day in the ICU, the red blood cell-platelet ratio was negatively correlated with Fibrinogen (cor = −0.29) (Fig 1D). These results indicate that an increased red blood cell-platelet ratio increases the risk of DIC in patients with gastrointestinal perforation complicated by sepsis.

### Modeling the optimal trajectory

**Fig 2:**
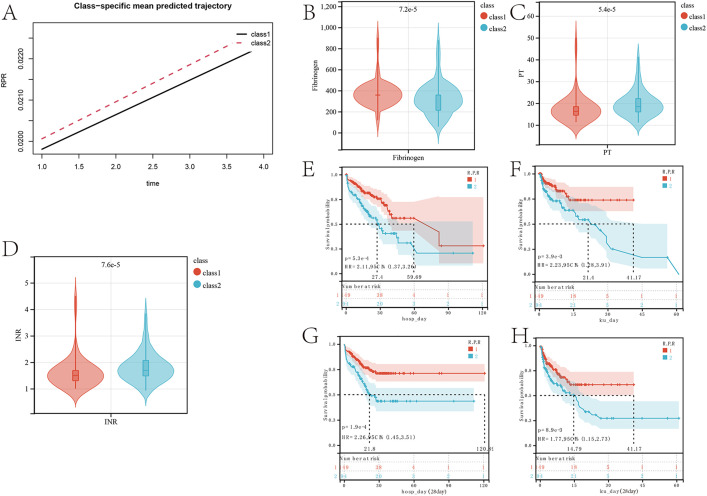
Impact of red blood cell-platelet ratio trajectories on prognosis and clinical outcomes in the first four days before ICU admission in the training set. **(A)** Impact trajectory categories of red blood cell-platelet ratio in the first four days before ICU admission in the training set. **(B)** Differences in fibrinogen levels between different impact trajectory categories of red blood cell-platelet ratio in the first four days before ICU admission in the training set. **(C)** (Differences in PT levels between different impact trajectory categories of red blood cell-platelet ratio in the first four days before ICU admission in the training set. **(D)** Differences in INR levels between different impact trajectory categories of red blood cell-platelet ratio in the first four days before ICU admission in the training set. **(E)** Survival curve analysis during hospitalization based on different red blood cell-platelet ratio trajectory categories. **(F)** Survival curve analysis during ICU stay based on different red blood cell-platelet ratio trajectory categories. **(G)** Survival curve analysis of 28-day prognosis based on different red blood cell-platelet ratio trajectory categories during hospitalization. **(H).** Survival curve analysis of 28-day prognosis based on different red blood cell-platelet ratio trajectory categories in the ICU.

The model with the smallest Bayesian Information Criterion (BIC) is selected as the best model, where the class 2 trajectory model has the smallest BIC value, suggesting that the model fits the data optimally. Platelet and red blood cell counts in patients vary over time, resulting in changes in the red blood cell-platelet ratio for each patient. Different trajectories of this ratio reflect changes in the physiological status of the patients. Subsequent trajectory analysis of this ratio revealed two distinct patterns in the red blood cell to platelet ratio for these patients (Fig 2A).

Clinical baseline data of patients with two different trajectory patterns show that, compared to class 2 patients, class 1 patients have higher body temperature, correspondingly higher red blood cell and platelet counts, but lower levels of Creatinine, PTT, and INR (Table 1). compared to the class2 group, the class1 group had higher Fibrinogen levels (Fig 2B), while PT (Fig 2C) and INR (Fig 2D) levels were lower. Compared to patients in class 2, patients in class 1 had a better prognosis during hospitalization (p = 5.3e-4, HR = 2.11) (Fig 2E), with a lower mortality rate at 28 days of hospitalization (p = 3.9e-3, HR = 2.23) (Fig 2F). In the ICU, patients in class 1 also had a better prognosis during ICU stay (p = 1.9e-4, HR = 2.26) (Fig 2G), and a lower mortality rate at 28 days in the ICU (p = 8.9e-3, HR = 1.77) (Fig 2H).

**Table 1:** Clinical baseline data.

Variable	Overall, N = 243^1^	class1, N = 149^1^	class2, N = 94^1^	p-value^2^
Age	67 (59, 78)	67 (59, 79)	66 (56, 78)	0.4
Gender				0.2
F	111 (46%)	73 (49%)	38 (40%)	
M	132 (54%)	76 (51%)	56 (60%)	
Weight	80 (66, 98)	80 (66, 97)	84 (67, 100)	0.3
Hypertension	104 (43%)	64 (43%)	40 (43%)	>0.9
Diabetes	57 (23%)	41 (28%)	16 (17%)	0.060
Malignant tumor	57 (23%)	31 (21%)	26 (28%)	0.2
Hyperlipidemia	75 (31%)	46 (31%)	29 (31%)	>0.9
Chronic bronchitis	23 (9.5%)	16 (11%)	7 (7.4%)	0.4
Heart rate	96 (84, 111)	93 (82, 111)	98 (87, 110)	0.13
Respiratory rate	20.4 (17.4, 23.7)	19.9 (17.2, 23.5)	20.6 (18.1, 24.8)	0.090
Temperature	36.91 (36.66, 37.22)	36.93 (36.69, 37.25)	36.84 (36.54, 37.11)	0.029
Systolic blood pressure	106 (97, 112)	106 (98, 114)	106 (97, 108)	0.3
Diastolic blood pressure	59 (54, 64)	59 (54, 65)	59 (53, 62)	0.6
WBC	12 (6, 19)	13 (8, 21)	9 (4, 16)	0.002
RBC	3.52 (2.96, 4.14)	3.46 (2.94, 4.11)	3.69 (2.99, 4.16)	0.3
Platelet	217 (146, 305)	267 (206, 358)	134 (85, 188)	<0.001
Hemoglobin	10.50 (8.70, 12.50)	10.30 (8.70, 12.40)	10.90 (8.72, 12.78)	0.3
PH	7.32 (7.24, 7.39)	7.33 (7.27, 7.39)	7.31 (7.21, 7.42)	0.2
PCO_2_	40 (34, 44)	40 (35, 44)	39 (33, 45)	0.5
PO_2_	113 (69, 158)	113 (66, 152)	114 (74, 170)	0.7
SpO_2_	97.15 (95.51, 98.48)	97.17 (95.91, 98.30)	97.15 (94.93, 98.72)	0.5
Sodium	138 (135, 142)	138 (135, 141)	139 (136, 142)	0.083
Potassium	4.10 (3.65, 4.55)	4.10 (3.70, 4.60)	4.05 (3.60, 4.40)	0.2
Calciumtotal	7.70 (7.20, 8.30)	7.70 (7.30, 8.30)	7.80 (7.00, 8.47)	>0.9
Glucose	135 (108, 162)	135 (111, 161)	132 (102, 166)	0.5
PTT	34 (30, 44)	32 (29, 41)	40 (33, 48)	<0.001
INR	1.50 (1.30, 1.80)	1.50 (1.30, 1.67)	1.67 (1.30, 1.98)	0.007
Total bilirubintotal	1.20 (0.60, 2.26)	1.00 (0.50, 2.26)	1.75 (0.80, 2.95)	0.001
Creatinine	1.20 (0.75, 1.95)	1.10 (0.70, 1.80)	1.45 (1.00, 2.30)	<0.001

^1^Median (IQR); n (%)

^2^Wilcoxon rank sum test; Pearson’s Chi-squared test

### Trajectory analysis of independent validation sets

**Fig 3:**
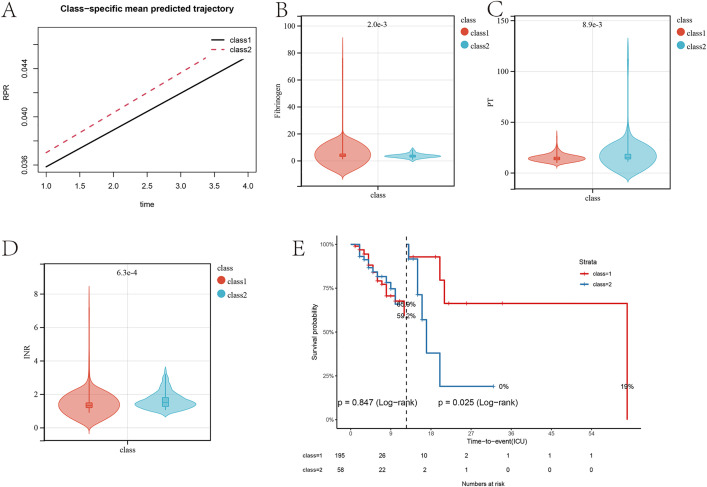
Impact of red blood cell-platelet ratio trajectories in the first four days before ICU admission on prognosis and clinical outcomes in the validation set. **(A)** Trajectory categories of the impact of red blood cell-platelet ratio in the first four days before ICU admission in the validation set. **(B)** Differences in fibrinogen levels between different trajectory categories of the impact of red blood cell-platelet ratio in the first four days before ICU admission in the validation set. **(C)** Differences in PT levels between different trajectory categories of the impact of red blood cell-platelet ratio in the first four days before ICU admission in the validation set. **(D)** Differences in INR levels between different trajectory categories of the impact of red blood cell-platelet ratio in the first four days before ICU admission in the validation set. **(E)** Analysis of survival curves during ICU stay based on different trajectory categories of the impact of red blood cell-platelet ratio in the first four days before ICU admission in the validation set (landmark survival).

Trajectory division of samples in the validation set (Fig 3A) was then followed by statistical analysis of the clinical indicators between the two groups. The results showed that: In the validation set, compared to the class2 group, the class1 group had higher Fibrinogen levels (Fig 3B), while PT (Fig 3C) and INR (Fig 3D) levels were lower. Survival analysis revealed that in the ICU, the 12-day prognosis was better in the class1 group (Fig 3E). These results confirm that the conclusions in the training set and validation set are consistent. The trajectory of the red blood cell-platelet ratio is more closely related to coagulation in patients with gastrointestinal perforation complicated by sepsis and impacts prognosis.

### Changes in the red blood cell-platelet ratio trajectory affect the prognosis of patients with gastrointestinal perforation complicated by sepsis

**Fig 4:**
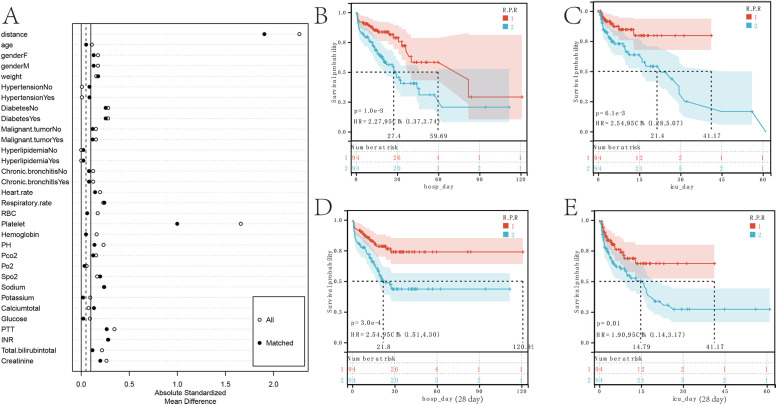
Prognostic Impact of Red Blood Cell-Platelet Ratio Trajectory Categories after PSM. **(A)** Covariate imbalance plot of propensity score matching. **(B)** Survival curve analysis during hospitalization based on different red blood cell-platelet ratio trajectory categories. **(C)** Survival curve analysis during ICU stay based on different red blood cell-platelet ratio trajectory categories. **(D)** Survival curve analysis of 28-day prognosis based on different red blood cell-platelet ratio trajectory categories during hospitalization. **(E)** Survival curve analysis of 28-day prognosis based on different red blood cell-platelet ratio trajectory categories in the ICU.

To further validate the impact of the red blood cell-platelet ratio trajectory on prognosis, a 1:1 matching of patients with the two trajectory patterns was conducted using propensity score matching (PSM), aiming to reduce the influence of other clinical factors on the conclusions of the prognostic analysis (Fig 4A). Clinical baseline data after PSM matching showed that, compared to patients in class 2, patients in class 1 had higher platelet counts, but lower levels of creatinine, PTT, and INR (Table 2). This outcome was consistent with the findings before matching.

**Table 2:** Clinical baseline data after PSM.

Variable	Overall, N = 188^1^	1, N = 94^1^	2, N = 94^1^	*p*-value^2^
Age	66 (58, 78)	66 (59, 79)	66 (56, 78)	0.7
Gender				0.4
F	82 (44%)	44 (47%)	38 (40%)	
M	106 (56%)	50 (53%)	56 (60%)	
Weight	82 (66, 98)	80 (63, 96)	84 (67, 100)	0.3
Hypertension	76 (40%)	36 (38%)	40 (43%)	0.6
Diabetes	41 (22%)	25 (27%)	16 (17%)	0.11
Malignant tumor	47 (25%)	21 (22%)	26 (28%)	0.4
Hyperlipidemia	59 (31%)	30 (32%)	29 (31%)	0.9
Chronic bronchitis	16 (8.5%)	9 (9.6%)	7 (7.4%)	0.6
Heart rate	97 (85, 111)	94 (82, 112)	98 (87, 110)	0.3
Respiratory rate	20.5 (17.4, 23.6)	20.2 (17.1, 23.5)	20.6 (18.1, 24.8)	0.13
Temperature	36.86 (36.64, 37.21)	36.89 (36.68, 37.22)	36.84 (36.54, 37.11)	0.2
Systolic blood pressure	106 (97, 112)	106 (97, 114)	106 (97, 108)	0.6
Diastolic blood pressure	59 (54, 64)	59 (54, 65)	59 (53, 62)	0.3
WBC	11 (5, 18)	12 (6, 19)	9 (4, 16)	0.079
RBC	3.63 (3.01, 4.16)	3.59 (3.05, 4.14)	3.69 (2.99, 4.16)	0.8
Platelet	189 (124, 251)	226 (189, 281)	134 (85, 188)	<0.001
Hemoglobin	10.80 (8.80, 12.70)	10.80 (8.93, 12.57)	10.90 (8.72, 12.78)	0.9
PH	7.32 (7.22, 7.39)	7.32 (7.25, 7.38)	7.31 (7.21, 7.42)	0.6
PCO_2_	39 (34, 45)	39 (35, 45)	39 (33, 45)	0.7
PO_2_	113 (69, 170)	112 (63, 158)	114 (74, 170)	0.7
SpO_2_	97.16 (95.34, 98.55)	97.28 (96.18, 98.41)	97.15 (94.93, 98.72)	0.4
Sodium	138 (135, 142)	138 (135, 141)	139 (136, 142)	0.075
Potassium	4.00 (3.60, 4.40)	4.00 (3.60, 4.47)	4.05 (3.60, 4.40)	>0.9
Calciumtotal	7.80 (7.00, 8.30)	7.80 (7.10, 8.20)	7.80 (7.00, 8.47)	0.5
Glucose	137 (112, 166)	142 (120, 168)	132 (102, 166)	0.11
PTT	35 (31, 44)	33 (29, 41)	40 (33, 48)	0.002
INR	1.50 (1.30, 1.90)	1.50 (1.23, 1.69)	1.67 (1.30, 1.98)	0.025
Total bilirubintotal	1.40 (0.78, 2.40)	1.30 (0.63, 2.26)	1.75 (0.80, 2.95)	0.085
Creatinine	1.30 (0.80, 2.00)	1.10 (0.70, 1.90)	1.45 (1.00, 2.30)	0.005

^1^Median (IQR); n (%)

^2^Wilcoxon rank su test; Pearson’s Chisquared test

Survival analysis was performed on the matched data. Compared to patients in class 2, patients in class 1 had a better prognosis during hospitalization (p = 1.0e-3, HR = 2.27) (Fig 4B), and a lower mortality rate at 28 days of hospitalization (p = 3e-4, HR = 2.54) (Fig 4D). In the ICU, patients in class 1 also had a better prognosis during their ICU stay (p = 6.1e-3, HR = 2.54) (Fig 4C), with a lower mortality rate at 28 days in the ICU (p = 0.01, HR = 1.9) (Fig 4E). These results demonstrate that the trajectory of the red blood cell-platelet ratio impacts the prognosis of patients with gastrointestinal perforation complicated by sepsis.

### Survival analysis

**Fig 5:**
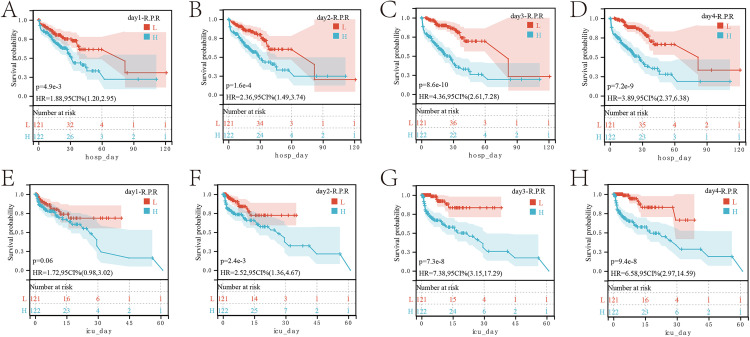
The red blood cell-platelet ratio affects prognosis. **(A)** Survival curve analysis of the red blood cell-platelet ratio on the first day in the ICU during hospitalization. **(B)** Survival curve analysis of the red blood cell-platelet ratio on the second day in the ICU during hospitalization. **(C)** Survival curve analysis of the red blood cell-platelet ratio on the third day in the ICU during hospitalization. **(D)** Survival curve analysis of the red blood cell-platelet ratio on the fourth day in the ICU during hospitalization. **(E)** Survival curve analysis of the red blood cell-platelet ratio on the first day in the ICU during ICU stay. **(F)** Survival curve analysis of the red blood cell-platelet ratio on the second day in the ICU during ICU stay. **(G)** Survival curve analysis of the red blood cell-platelet ratio on the third day in the ICU during ICU stay. **(H)** Survival curve analysis of the red blood cell-platelet ratio on the fourth day in the ICU during ICU stay.

Correspondingly, the red blood cell-platelet ratio affects the prognosis. On the first day in the ICU, patients with a high red blood cell-platelet ratio had a worse prognosis during hospitalization (p = 4.9e-3, HR = 1.88) and in the ICU (p = 0.06, HR = 1.72) (Fig 5A, 5E). On the second day in the ICU, patients with a high red blood cell-platelet ratio had a worse prognosis during hospitalization (p = 1.6e-4, HR = 2.36) and in the ICU (p = 2.4e-3, HR = 2.52) (Fig 5B, 5F). By the third day in the ICU, patients with a high red blood cell-platelet ratio had a worse prognosis during hospitalization (p = 8.6e-10, HR = 4.36) and in the ICU (p = 7.3e-8, HR = 7.38) (Fig 5C, 5G). On the fourth day in the ICU, patients with a high red blood cell-platelet ratio also had a worse prognosis during hospitalization (p = 7.2e-9, HR = 3.89) and in the ICU (p = 9.4e-8, HR = 6.58) (Fig 5D, 5H). These results indicate that the relationship between the red blood cell-platelet ratio and prognosis is stable and reliable.

### Analysis of the red blood cell-platelet ratio trajectory

**Fig 6:**
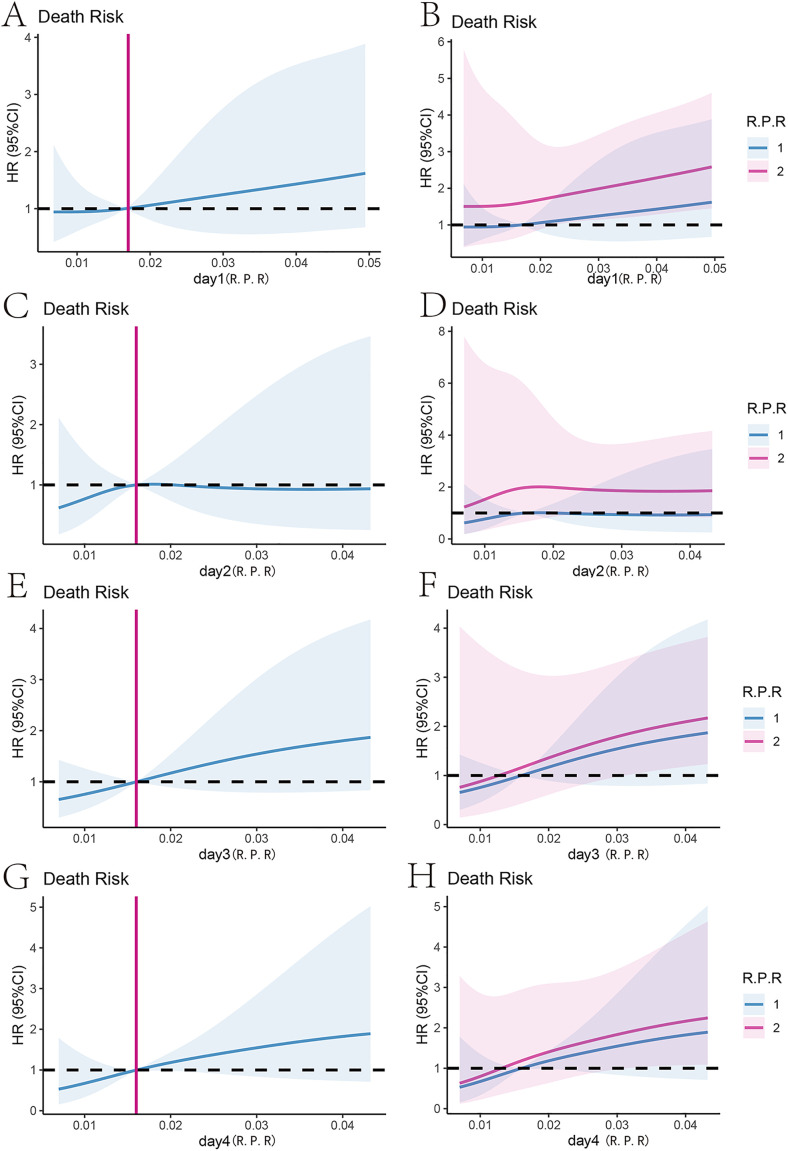
Red blood cell-platelet ratio and HR-restriction cube diagram during hospitalization. **(A)** Relationship between red blood cell-platelet ratio and HR during hospitalization on the first day in the ICU. **(B)** Relationship between red blood cell-platelet ratio and HR during hospitalization on the first day in the ICU for different trajectory groups. **(C)** Relationship between red blood cell-platelet ratio and HR during hospitalization on the second day in the ICU. **(D)** Relationship between red blood cell-platelet ratio and HR during hospitalization on the second day in the ICU for different trajectory groups. **(E)** Relationship between red blood cell-platelet ratio and HR during hospitalization on the third day in the ICU. **(F)** Relationship between red blood cell-platelet ratio and HR during hospitalization on the third day in the ICU for different trajectory groups. **(G)** Relationship between red blood cell-platelet ratio and HR during hospitalization on the fourth day in the ICU. **(H)** Relationship between red blood cell-platelet ratio and HR during hospitalization on the fourth day in the ICU for different trajectory groups.

Restrictive cubic spline graph results show that the risk of death in patients with gastrointestinal perforation complicated by sepsis increases with the rise in the red blood cell-platelet ratio, a trend that is consistent in the four days before ICU admission (Fig 6A, 6C, 6E, 6G). Stratifying patients based on the trajectory of the red blood cell-platelet ratio reveals that patients with class 2 have a higher risk of death compared to those in class 1 with the same red blood cell-platelet ratio (Fig 6B,6D, 6F, 6H). This outcome suggests that identifying the trajectory of the red blood cell-platelet ratio in patients with gastrointestinal perforation complicated by sepsis is beneficial for further prognostic predictions.

### Red blood cell-platelet ratio trajectory associated with MIMIC-IVprognosis nomogram

**Fig 7:**
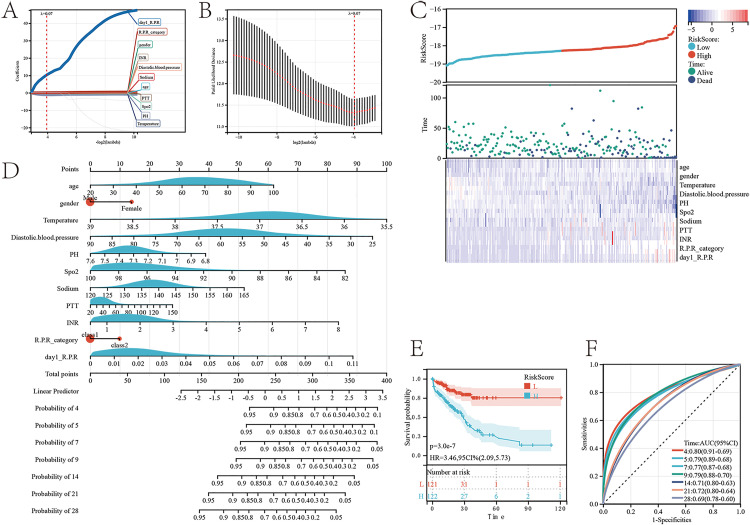
Prognostic model for the red blood cell-platelet ratio and its trajectory categories during hospitalization. **(A)** LASSO Regression Coefficient Path Plot of Risk Factors. **(B)** Cross-Validation Curve. **(C)** Risk Factor Correlation Plot. **(D)** Predicted Probability Plot of Risk of Death during Hospitalization. **(E)** Score Survival Curve Plot. **(F)** Predicted ROC Curve for the 4, 5, 7, 9, 14, 21, and 28-day prognostic timelines during hospitalization.

Univariate analysis was performed for all variables (Table 3) to conduct preliminary screening. Using LASSO regression analysis, with λ._min_ (= 0.06) identified as the optimal λ value, prognostic indicators for patients with gastrointestinal perforation complicated by sepsis were obtained, including age, gender, temperature, diastolic blood pressure, pH, Spo2, sodium, PTT, INR, R.P.R_category, and day1_R.P.R. Among these, day1_R.P.R exhibits the highest predictive power (Fig 7A,7B). The risk factor linkage diagram shows that these variables are significantly associated with the inpatient prognosis of patients with gastrointestinal perforation complicated by sepsis (Fig 7C).

**Table 3:** Univariate analysis of hospitalized patients.

name		All	HR (univariable)
Gender	M	132 (54.3%)	
	F	111 (45.7%)	1.69 (1.10–2.62, p = .018)
Diabetes	No	186 (76.5%)	
	Yes	57 (23.5%)	0.77 (0.45–1.32, p = .348)
Hypertension	No	139 (57.2%)	
	Yes	104 (42.8%)	0.95 (0.61–1.48, p = .811)
Malignant tumor	No	186 (76.5%)	
	Yes	57 (23.5%)	1.37 (0.85–2.20, p = .200)
Hyperlipidemia	No	168 (69.1%)	
	Yes	75 (30.9%)	0.84 (0.51–1.37, p = .478)
Chronic bronchitis	No	220 (90.5%)	
	Yes	23 (9.5%)	0.81 (0.37–1.77, p = .593)
RPR class	1	149 (61.3%)	
	2	94 (38.7%)	2.11 (1.37–3.26, p < .001)
Age	Mean ± SD	67.1 ± 14.2	1.02 (1.01–1.04, p = .007)
Weight	Mean ± SD	84.8 ± 26.0	1.00 (0.99–1.01, p = .945)
Heartrate	Mean ± SD	97.0 ± 18.2	1.01 (1.00–1.02, p = .226)
Respiratory rate	Mean ± SD	20.7 ± 4.4	1.04 (0.99–1.09, p = .100)
Temperature	Mean ± SD	37.0 ± 0.5	0.47 (0.30–0.76, p = .002)
Systolic blood pressure	Mean ± SD	105.7 ± 14.6	0.99 (0.97–1.00, p = .156)
Diastolic blood pressure	Mean ± SD	58.9 ± 10.1	0.97 (0.95–0.99, p = .003)
WBC	Mean ± SD	14.3 ± 11.0	1.01 (0.99–1.03, p = .399)
RBC	Mean ± SD	3.6 ± 0.9	0.87 (0.68–1.10, p = .236)
Platelet	Mean ± SD	239.2 ± 142.7	1.00 (1.00–1.00, p = .041)
Hemoglobin	Mean ± SD	10.7 ± 2.5	0.95 (0.87–1.03, p = .209)
PH	Mean ± SD	7.3 ± 0.1	0.05 (0.01–0.22, p < .001)
PCO_2_	Mean ± SD	41.4 ± 13.1	1.00 (0.99–1.02, p = .578)
PO_2_	Mean ± SD	136.9 ± 95.8	1.00 (1.00–1.00, p = .667)
SpO2	Mean ± SD	96.7 ± 2.5	0.94 (0.87–1.00, p = .049)
Sodium	Mean ± SD	138.3 ± 5.2	1.06 (1.01–1.10, p = .011)
Potassium	Mean ± SD	4.2 ± 0.8	0.98 (0.72–1.33, p = .884)
Calciumtotal	Mean ± SD	7.8 ± 1.0	1.01 (0.82–1.23, p = .934)
Glucose	Mean ± SD	145.3 ± 69.2	1.00 (1.00–1.01, p = .267)
PTT	Mean ± SD	40.5 ± 20.6	1.01 (1.00–1.02, p = .031)
INR	Mean ± SD	1.7 ± 0.7	1.35 (1.07–1.69, p = .010)
Total bilirubintotal	Mean ± SD	2.3 ± 3.5	1.02 (0.98–1.07, p = .276)
Creatinine	Mean ± SD	1.6 ± 1.5	1.00 (0.89–1.12, p = .986)
day1	Mean ± SD	0.0 ± 0.0	1578789983.84 (30260.78–82369913017388.94, p < .001)
day2	Mean ± SD	0.0 ± 0.0	53009.16 (34.16–82265663.99, p = .004)
day3	Mean ± SD	0.0 ± 0.0	36252.50 (7.24–181460470.87, p = .016)
day4	Mean ± SD	0.0 ± 0.0	427.80 (1.94–94157.23, p = .028)

n=243, events=83, Likelihood ratio test=85.79 on 34 df (p<.001)

Subsequently, based on the above variables, a prognostic nomogram for inpatients with gastrointestinal perforation complicated by sepsis was constructed. By utilizing the clinical values for each patient, this nomogram enables the estimation of the survival probability during a patient’s hospital stay at 4, 5, 7, 9, 14, 21, and 28 days (Fig 7D). Patients with lower risk scores have a better prognosis (p = 3e-7, HR = 3.46) (Fig 7E). The accuracy of the nomogram prediction was evaluated using ROC curves, with AUC values of 0.8 for 4 days, 0.79 for 5 days, 0.77 for 7 days, 0.79 for 9 days, 0.71 for 14 days, 0.72 for 21 days, and 0.69 for 28 days (Fig 7F).

### Red blood cell-platelet ratio trajectory related to external validation prognosis nomogram

**Fig 8:**
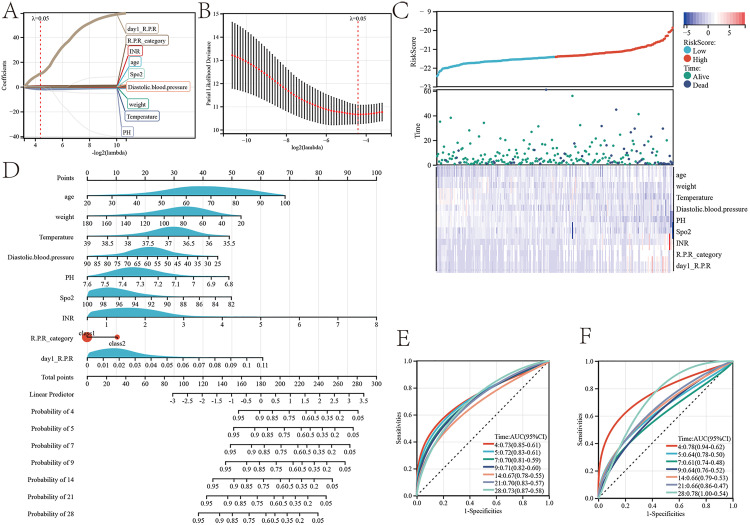
Prognostic model for the red blood cell-platelet ratio and its trajectory categories during ICU stay. **(A)** LASSO Regression Coefficient Path Plot of Risk Factors. **(B)** Cross-Validation Curve.© Risk Factor Correlation Plot. **(D)** Predicted Probability Plot of Risk of Death during ICU stay. **(E)** Score Survival Curve Plot. **(F)** Predicted ROC Curve for the 4, 5, 7, 9, 14, 21, and 28-day prognostic timelines during ICU stay.

Univariate analysis was performed for all variables (Table 4) to conduct preliminary screening. Related variables were included in the LAASO regression and λ_min_ was selected as the best λ value. When λ_min_ = 0.05, prognostic indicators for patients with gastrointestinal perforation complicated by sepsis in the ICU were obtained, including age, weight, temperature, diastolic blood pressure, pH, Spo2, INR, R.P.R_category, and day1_R.P.R Among these, day1_R.P.R exhibits the highest predictive power (Fig 8A–8B). The risk factor linkage diagram demonstrates a significant correlation between these variables and the ICU prognosis of patients with gastrointestinal perforation complicated by sepsis (Fig 8C).

**Table 4:** Univariate analysis of ICU patients.

Name		All	HR (univariable)
Gender	F	111 (45.7%)	
	M	132 (54.3%)	0.78 (0.46-1.33, p=.366)
Hypertension	No	139 (57.2%)	
	Yes	104 (42.8%)	1.00 (0.58-1.73, p=.994)
Diabetes	No	186 (76.5%)	
	Yes	57 (23.5%)	0.70 (0.35-1.40, p=.314)
Malignant tumor	No	186 (76.5%)	
	Yes	57 (23.5%)	1.42 (0.78-2.58, p=.256)
Hyperlipidemia	No	168 (69.1%)	
	Yes	75 (30.9%)	0.74 (0.40-1.39, p=.347)
Chronic bronchitis	No	220 (90.5%)	
	Yes	23 (9.5%)	0.89 (0.35-2.25, p=.809)
RPR class	1	149 (61.3%)	
	2	94 (38.7%)	2.23 (1.28-3.91, p=.005)
Age	Mean ± SD	67.1 ± 14.2	1.03 (1.01-1.05, p=.010)
Weight	Mean ± SD	84.8 ± 26.0	0.98 (0.96-0.99, p=.021)
Heartrate	Mean ± SD	97.0 ± 18.2	1.01 (0.99-1.02, p=.306)
Respiratory rate	Mean ± SD	20.7 ± 4.4	1.04 (0.98-1.10, p=.207)
Temperature	Mean ± SD	37.0 ± 0.5	0.46 (0.26-0.82, p=.008)
Systolic blood pressure	Mean ± SD	105.7 ± 14.6	0.98 (0.96-1.00, p=.116)
Diastolic blood pressure	Mean ± SD	58.9 ± 10.1	0.97 (0.94-1.00, p=.021)
WBC	Mean ± SD	14.3 ± 11.0	0.99 (0.96-1.01, p=.298)
RBC	Mean ± SD	3.6 ± 0.9	0.80 (0.59-1.08, p=.148)
Platelet	Mean ± SD	239.2 ± 142.7	1.00 (1.00-1.00, p=.048)
Hemoglobin	Mean ± SD	10.7 ± 2.5	0.94 (0.84-1.05, p=.257)
PCO_2_	Mean ± SD	41.4 ± 13.1	1.01 (0.99-1.03, p=.387)
PH	Mean ± SD	7.3 ± 0.1	0.03 (0.00-0.19, p<.001)
PO_2_	Mean ± SD	136.9 ± 95.8	1.00 (1.00-1.00, p=.968)
SpO_2_	Mean ± SD	96.7 ± 2.5	0.92 (0.86-0.98, p=.006)
Sodium	Mean ± SD	138.3 ± 5.2	1.04 (0.98-1.09, p=.171)
Potassium	Mean ± SD	4.2 ± 0.8	1.20 (0.83-1.72, p=.334)
Calciumtotal	Mean ± SD	7.8 ± 1.0	0.83 (0.62-1.10, p=.189)
Glucose	Mean ± SD	145.3 ± 69.2	1.00 (1.00-1.01, p=.725)
PTT	Mean ± SD	40.5 ± 20.6	1.01 (1.00-1.02, p=.050)
INR	Mean ± SD	1.7 ± 0.7	1.42 (1.15-1.76, p=.001)
Total bilirubintotal	Mean ± SD	2.3 ± 3.5	1.06 (0.99-1.14, p=.093)
Creatinine	Mean ± SD	1.6 ± 1.5	1.01 (0.86-1.18, p=.918)
day1	Mean ± SD	0.0 ± 0.0	128639055.75 (877.19-18864784143430.58, p=.002)
day2	Mean ± SD	0.0 ± 0.0	2869.13 (1.00-8251131.18, p=.050)
day3	Mean ± SD	0.0 ± 0.0	76.86 (0.00-1912737.83, p=.400)
day4	Mean ± SD	0.0 ± 0.0	26.61 (0.05-14765.54, p=.309)

n=243, events=55, Likelihood ratio test=82.45 on 34 df(p<.001)

Subsequently, based on the above variables, a prognostic nomogram for patients with gastrointestinal perforation complicated by sepsis in the ICU was constructed. By using the clinical values of each patient, this nomogram enables the estimation of the survival probability for each patient during their ICU stay at 4, 5, 7, 9, 14, 21, and 28 days (Fig 8D). The accuracy of the nomogram prediction was evaluated using ROC curves, with AUC values of 0.73 for 4 days, 0.72 for 5 days, 0.7 for 7 days, 0.71 for 9 days, 0.67 for 14 days, 0.7 for 21 days, and 0.73 for 28 days (Fig 8E). A total of 253 patients were included in this study as an independent external validation test (Table 5). In the validation set, the accuracy of the nomogram prediction was evaluated using ROC curves, with AUC values of 0.78 for 4 days, 0.64 for 5 days, 0.61 for 7 days, 0.64 for 9 days, 0.66 for 14 days, 0.66 for 21 days, and 0.78 for 28 days (Fig 8F).

**Table 5:** Baseline data of clinical ICU patients.

name		class1 (N = 195)	class2 (N = 58)	total (N = 253)	p
PH	Mean ± SD	7.4 ± 0.1	7.3 ± 0.1	7.4 ± 0.1	0.048
INR	Median (IQR)	1.3 (1.2 to 1.5)	1.5 (1.2 to 1.8)	1.3 (1.2 to 1.6)	<.001
SpO2	Median (IQR)	0.3 (0.2 to 0.4)	0.2 (0.2 to 0.3)	0.3 (0.2 to 0.4)	0.001
Age	Median (IQR)	65.0 (54.0 to 76.5)	64.0 (57.0 to 72.0)	65.0 (55.0 to 76.0)	0.714
Weight	Median (IQR)	65.0 (56.0 to 70.0)	62.0 (56.0 to 68.0)	63.0 (56.0 to 69.0)	0.429
Temperature	Median (IQR)	38.4 (38.4 to 38.6)	38.4 (38.3 to 38.5)	38.4 (38.3 to 38.6)	0.223
DAY1RPR	Median (IQR)	0.0 (0.0 to 0.0)	0.1 (0.0 to 0.1)	0.0 (0.0 to 0.0)	<.001

## Discussion

This study has revealed that the red blood cell-platelet ratio influences the prognosis of patients with sepsis following gastrointestinal perforation during hospitalization and in the ICU. Moreover, changes in the trajectory of the red blood cell-platelet ratio also impact patient outcomes. For patients in the acute phase of the disease, the counts of red blood cells and platelets are somewhat influenced by fluid volume status. By utilizing the red blood cell-platelet ratio, a precise integration of information from both parameters can be achieved, effectively leveraging data on blood volume and platelets.

Previous studies have shown that platelet trajectories are independent prognostic factors for outcomes, separate from thrombocytopenia. In the absence of thrombocytopenia, platelet trajectories can also impact patient survival rates [29]. Therefore, conducting trajectory analysis on clinical indicators is beneficial for uncovering their potential clinical significance. Latent class analysis (LCA) has enabled epidemiologists to overcome the practical constraints faced by traditional diagnostic test evaluation methods [30]. Trajectory analysis is a longitudinal data analysis method that models irregular longitudinal measurements as polynomial functions of time, determining dynamic trends of biomarkers over a period to detect high-risk patients early for improved prognosis. Trajectory modelling techniques have been developed to identify subgroups within populations and better understand intra- and inter-individual variability in health outcomes over time [31]. LCA allows the reuse of repeated measurement data to identify trajectories of individual variable changes, overcoming limitations of single time-point variables to enhance predictive accuracy in research [32,33]. Our study utilized to analyze the trajectory development of the red blood cell-platelet ratio in patients with gastrointestinal perforation and sepsis admitted to the ICU, revealing two distinct trajectories. Patients on different trajectories showed significant differences in their prognosis, a previously unexplored aspect. Therefore, medical professionals should pay attention to this indicator in clinical practice to enhance patient management efficiency and improve prognosis.

Sepsis is characterized by decreased count of RBC and platelet, but reduced RBC count has no diagnostic or prognostic power for sepsis, platelet count and trajectory is useful diagnostic and prognostic biomarker in sepsis [34–38].Changes in PER are indicative of the balance between erythrocytes and platelet counts. We found the index of PER was associated with prognosis of abdominal septic patients underwent emergent surgery procedure. Previous studies have explored the prognostic impact of the platelet-to-red blood cell ratio on patients with bleeding receiving blood transfusions. Compared to trauma patients with lower PLT/RBC ratios, those with higher PLT/RBC ratios exhibit significantly lower mortality rates at 24 hours, and 28–30 days [25]. Our study focuses on the impact of the red blood cell to platelet ratio on the prognosis of patients with sepsis in the gastrointestinal tract. The lower the red blood cell to platelet ratio, the better the prognosis of the patients. This trend is consistent with the trend observed in the study mentioned above. The value of PER has also been shown that platelets inhibited the parasite’s invasion under the condition of Plasmodium falciparum infection below physiological ratios [39]. These results indicate that the ratio of red blood cells to platelets is a sensitive indicator of patients’ health status and disease prognosis.

Literature data shows organ dysfunction in the septic patients is associated with prognosis [40–42]. During the course of the disease, the number of blood platelets will change secondarily [43–45] Our study found that the ratio of red blood cells to platelets is closely related to coagulation indicators, affecting not only clotting time but also closely related to fibrinogen concentration. These findings suggest that this indicator is closely associated with the occurrence of disseminated intravascular coagulation (DIC). Furthermore, this ratio also affects blood creatinine concentration, therefore, it can also reflect the extent of renal function damage in patients. These may be reasons why the red cell-platelet ratio influences patient prognosis. In addition, our study found that the different trajectories of PER are closely related to these complications. Compared to class 2 patients, class 1 patients have higher body temperature, correspondingly higher red blood cell and platelet counts, but lower levels of Creatinine, PTT, and INR. Therefore, through early trajectory analysis and the clinical prognosis nomogram constructed in this study, individual differences can be identified earlier, leading to a more precise and efficient detection of high-risk patients. This is advantageous for early clinical intervention and improving patient survival rates. These results indicate to healthcare providers the necessity of dynamically monitoring the platelets and red blood cells of patients with gastrointestinal perforation complicated by sepsis, in order to obtain trajectory data that may not be easily detected.

This study demonstrates several advantages. First, platelet and red blood cell are all easy and common to measure, providing a simple tool for the early assessment of clinical condition and prognosis for septic patients. Second, we utilize machine learning to build model and further develop external validation in order to improve the credibility of the model. However, there are also some limitations. First, this is a retrospective cohort study, which may result in selective bias. Second, MIMIC-IV data comes from a single medical center, which may affect the representativeness of the subjects. Large-scale multicenter studies are needed to further validate the findings of this study.

## Conclusion

Our results suggested that red blood cell to platelet ratio might be a novel biomarker for prognosis of abdominal septic patients, influencing clotting and kidney function.

## Supporting information

S1 Table International Classification of Diseases Codes. ICD-9 and ICD-10 codes for diseases.
(XLSX)
